# Association of cancer mortality with postdiagnosis overweight and obesity using body mass index

**DOI:** 10.18632/oncotarget.6517

**Published:** 2015-12-09

**Authors:** Xinsen Xu, Lei Zhou, Runchen Miao, Wei Chen, Yanyan Zhou, Qing Pang, Kai Qu, Chang Liu

**Affiliations:** ^1^ Department of Hepatobiliary Surgery, The First Affiliated Hospital of Xi'an Jiaotong University, Xi'an 710061, China; ^2^ Department of Hepatobiliary Surgery, The Affiliated Hospital of Binzhou Medical University, Binzhou 256603, China

**Keywords:** cancer, prognosis, body mass index, overweight, obesity

## Abstract

Although overweight and obesity increase cancer risk, it is still controversial with respect to cancer mortality. In the current study, we enrolled 2670 patients of 14 tumor types from the Cancer Genome Atlas (TCGA) project, to identify the prognostic role of overweight and obesity in cancer patients. After dividing the patients into different groups by the body mass index (BMI), we found significant lower mortality in the obesity group. In addition, we also treat BMI value as a binary categorical variable or continuous variable, respectively. We found significant lower mortality in the higher BMI group. Furthermore, when focusing on each tumor type, cervical cancer and bladder cancer showed lower mortality in the patients with higher BMI values. Taken together, our results demonstrate that postdiagnosis obesity might indicate a better prognosis in cancer patients. However, these findings should be interpreted cautiously because of small sample size.

## INTRODUCTION

Prevalence of overweight and obesity has shown a significant increase during the last decades [1, 2]. Besides higher risk of cardiovascular disease and diabetes, evidence suggests that the excess body weight is also associated with higher cancer risk [3, 4]. However, it is still controversial with respect to cancer motality.

Body mass index (BMI) is a person's weight in kilograms divided by the square of height in meters. It is an attempt to quantify the amount of tissue mass (muscle, fat, and bone), and then categorize that individual as underweight, normal weight, overweight, or obese. By the analysis of more than 2.88 million individuals, Flegal *et al*. concluded that obesity was associated with higher mortality, while overweight was associated with lower mortality [5, 6]. However, they did not specify the cancer population, and little conclusion could be drawn when focusing on cancer patients.

The cancer genome atlas (TCGA) project motivated large-scale coordinated cancer genomic efforts to obtain complete catalogs of the genomic alterations in cancer. Besides the comprehensive molecular profiling of each tumor, it also provides valuable clinical data [7]. In the present study, we used the survival data of over 2000 cancer patients (14 tumor types) from the TCGA project, to identify the roles of overweight and obesity in the prognosis of cancer.

## RESULTS

### Patient demographics and outcomes

All TCGA cancer data were downloaded (34 tumor types, 11091 patients). However, only 14 tumor types (2670 patients) had the complete data of height, weight, and survival information [Uveal Melanoma (UVM), Uterine Corpus Endometrial Carcinoma (UCEC), Uterine Carcinosarcoma (UCS), Thymoma (THYM), Skin Cutaneous Melanoma (SKCM), Rectum adenocarcinoma (READ), Lymphoid Neoplasm Diffuse Large B-cell Lymphoma (DLBC), Liver hepatocellular carcinoma (LIHC), Kidney renal papillary cell carcinoma (KIRP), Esophageal carcinoma (ESCA), Colon adenocarcinoma (COAD), Cholangiocarcinoma (CHOL), Cervical squamous cell carcinoma and endocervical adenocarcinoma (CESC) and Bladder Urothelial Carcinoma (BLCA)]. Thus, 2670 patients of 14 tumor types were included for analysis.

Clinical characteristics were listed in Table 1. There were 1222 men and 1448 women. The mean age was 61.3, with the average height of 167 cm and average weight of 79.9 kg. Most patients received surgical therapy, since the TCGA project requires resected tissues for genome analysis. The median survival time was 102.4 months (95% CI, 92.7-114.7). At the time of analysis, 2195 patients were alive, and 475 patients were dead.

**Table 1:** Baseline characteristics of the cancer patients

Cancer Type	Age (mean±SD)	Gender (male/female)	Stage (I/II/III/IV/unavailable)	height (mean±SD)	weight (mean±SD)	BMI (mean±SD)
UVM	61.3±13.7	28/25	0/17/32/3/1	166.5±10.7	79.4±22.8	28.7±8.9
UCEC	63.7±11.1	0/515	322/49/115/29/0	161.2±8.2	87.7±25.4	33.9±12.1
UCS	69.5±9.2	0/52	22/5/16/9/0	157.7±7.7	73.3±20.2	29.6±9.0
THYM	59.1±13.0	53/47	30/51/13/6/0	167.3±11.1	76.8±19.6	27.3±6.1
SKCM	59.5±15.3	143/99	34/89/94/11/14	170.1±9.4	81.4±19.3	28.1±6.1
READ	60.5±11.7	44/31	9/20/31/13/2	170.7±9.6	79.1±22.5	26.9±5.8
DLBC	56.3±14.1	21/26	8/17/4/12/6	164.9±9.1	71.0±18.4	26.0±5.9
LIHC	59.5±12.8	231/105	163/77/76/4/16	167.4±10.7	73.0±19.6	26.2±8.4
KIRP	61.1±12.0	144/46	113/13/30/9/25	172.1±14.4	87.6±21.2	32.1±33.3
ESCA	62.3±11.7	151/24	18/73/53/9/22	172.1±8.6	75.1±19.1	25.3±5.9
COAD	64.3±13.1	124/108	32/93/76/25/6	168.4±12.3	81.2±20.2	29.4±17.2
CHOL	63.4±12.9	16/19	19/8/1/7/0	167.3±11.7	79.2±20.6	28.0±5.3
CESC	48.5±13.5	0/258	132/62/39/20/5	160.8±7.3	72.4±19.9	28.0±7.7
BLCA	67.7±10.5	267/93	2/117/119/120/2	171.6±10.2	80.3±21.1	27.1±6.2
**OVERALL**	61.3±13.4	1222/1448	904/691/699/277/99	167.0±11.0	79.9±22.0	29.0±13.1

UVM, Uveal Melanoma; UCEC, Uterine Corpus Endometrial Carcinoma; UCS, Uterine Carcinosarcoma; THYM, Thymoma; SKCM, Skin Cutaneous Melanoma; READ, Rectum adenocarcinoma; DLBC, Lymphoid Neoplasm Diffuse Large B-cell Lymphoma; LIHC, Liver hepatocellular carcinoma; KIRP, Kidney renal papillary cell carcinoma; ESCA, Esophageal carcinoma; COAD, Colon adenocarcinoma; CHOL, Cholangiocarcinoma; CESC, Cervical squamous cell carcinoma and endocervical adenocarcinoma; BLCA, Bladder Urothelial Carcinoma.

### Analyses of the cancer patients in the whole population

In this study, we applied the National Heart, Lung, and Blood Institute's BMI categories of underweight (BMI<18.5), normal weight (18.5 ≤ BMI < 25), overweight (25 ≤ BMI < 30), and obesity (BMI ≥ 30). Grade 1 obesity was defined as a BMI of 30 to less than 35; grade 2 obesity, a BMI of 35 to less than 40; and grade 3 obesity, a BMI of 40 or greater [5, 6].

**Figure 1:**
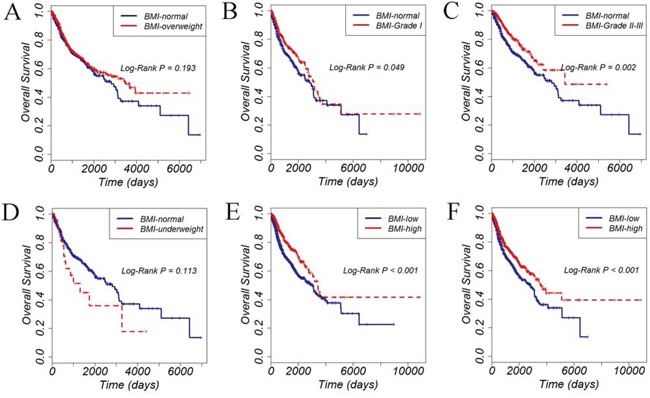
Overall survival of 2670 cancer patients stratified by different BMI groups **A.** overweight versus normal weight; **B.** Grade 1 obesity versus normal weight; **C.** Grade 2 and Grade 3 obesity versus normal weight; **D.** underweight versus normal weight; **E.** high BMI value versus low BMI value, with the BMI cutoff determined by ROC curve; **F.** high BMI value versus low BMI value, with the BMI cutoff determined by median value.

To gain insight into the prognostic role of obesity, we utilized the Kaplan-Meier curves stratified by different BMI groups. As shown in Figure 1A–1D, patients with grade 1, grade 2 and grade 3 obesity, showed significantly lower mortality (*P* < 0.05). We combined the grade 2 and grade 3 patients into one group, simply because the sample size is relatively small in grade 2 and grade 3. Although lower mortality was observed in overweight group, and higher mortality was observed in underweight group, they didn't reach statistical significance. In addition, cox regression analysis also confirmed the lower mortality in grade 1 obesity (HR=0.76, 95%CI, 0.58-0.99, *P* = 0.049), grade 2 and grade 3 obesity (HR=0.63, 95%CI, 0.46-0.85, *P* < 0.001), but not in the overweight group (HR=0.87, 95%CI, 0.70-1.08, *P* = 0.19), or in the underweight group (HR=1.50, 95%CI, 0.91-2.47, *P* = 0.115).

We then used the receiver operating characteristic (ROC) curves to determine the cutoff value of BMI. Thus, the BMI value of 31.2 (determined by the ROC curve) and 27.0 (median value)were used to explore the prognostic value of obesity, respectively. As shown in Figure 1E–1F, patients with higher BMI values showed lower mortality, no matter stratified by the ROC cutoff value (Figure 1E) or the median value (Figure 1F).

In univariable analysis, age (HR=1.60, 95%CI, 1.34-1.92, *P* < 0.001), tumor stage (HR=2.63, 95%CI, 2.18-3.17, *P* < 0.001), and the BMI values (HR=0.62, 95%CI, 0.49-0.77, *P* < 0.001) were significantly associated with the overall survival (Table 3). Multivariate analysis showed that age (HR=1.47, 95%CI, 1.22-1.77, *P* < 0.001), tumor stage (HR=2.52, 95%CI, 2.09-3.04, *P* < 0.001), and the BMI values (HR=0.64, 95%CI, 0.51-0.81, *P* < 0.001) were still significantly associated with the overall survival (Table 2).

**Table 2:** Univariate and multivariate analysis in the whole population

Clinical Variables	univariate analysis		multivariate analysis	
	HR(95%CI)	*P*	HR(95%CI)	*P*
Age(>=67 vs <67)	1.60(1.34-1.92)	**<0.001**	1.47(1.22-1.77)	**<0.001**
Gender(male vs female)	1.12(0.90-1.40)	0.33		
Stage(III-IV vs I-II)	2.63(2.18-3.17)	**<0.001**	2.52(2.09-3.04)	**<0.001**
BMI(high vs low)	0.62(0.49-0.77)	**<0.001**	0.64(0.51-0.81)	**<0.001**

**Table 3:** Cox regression analysis of patients in each cancer type

Cancer Type	Age (>=67 vs <67)		Gender (male vs female)		Stage (III-IV vs I-II)		BMI-ROC (high vs low)		BMI-median (high vs low)	
	HR (95%CI)	*P*	HR (95%CI)	*P*	HR (95%CI)	*P*	HR (95%CI)	*P*	HR (95%CI)	*P*
UVM	4.1(1.19–14.09)	**0.025**	1.51(0.44–5.17)	0.52	2.02(0.44–9.36)	0.37	–	–	1.30(0.39–4.29)	0.67
UCEC	1.58(0.86–2.87)	0.14	–	–	5.02(2.71–9.27)	**<0.001**	0.60(0.28–1.30)	0.19	0.68(0.37–1.25)	0.21
UCS	1.29(0.58–2.87)	0.53	–	–	1.95(0.88–4.31)	0.1	0.91(0.49–2.09)	0.83	1.04(0.46–2.34)	0.92
THYM	1.72(0.33–8.84)	0.52	0.45(0.074–2.75)	0.39	1.25(0.14–11.27)	0.84	0.25(0.029–2.18)	0.21	0.62(0.10–3.72)	0.6
SKCM	2.07(1.18–3.66)	**0.012**	1.60(0.87–2.95)	0.13	2.24(1.27–3.97)	**0.006**	1.42(0.76–2.66)	0.27	1.09(0.63–1.89)	0.76
READ	4.04(0.75–21.7)	0.1	1.71(0.36–8.15)	0.5	4.45(0.51–38.62)	0.18	2.52(0.48–13.12)	0.27	1.14(0.22–5.92)	0.88
DLBC	–	–	0.36(0.038–3.53)	0.38	3.05(0.31–29.88)	0.34	0.31(0.042–2.24)	0.24	0.59(0.081–4.33)	0.61
LIHC	1.57(1.01–2.46)	**0.049**	0.74(0.47–1.17)	0.2	1.55(0.92–2.59)	0.097	1.28(0.82–2.00)	0.28	1.01(0.64–1.58)	0.98
KIRP	0.83(0.31–2.25)	0.73	0.61(0.21–1.73)	0.35	5.87(2.07–16.62)	**<0.001**	0.39(0.15–1.02)	0.054	0.53(0.20–1.43)	0.21
ESCA	1.05(0.61–1.81)	0.86	1.80(0.65–5.02)	0.26	2.08(1.18–3.69)	**0.012**	0.74(0.41–1.35)	0.33	0.67(0.38–1.17)	0.15
COAD	1.26(0.54–2.94)	0.59	1.47(0.65–3.35)	0.36	1.88(0.83–4.28)	0.13	0.36(0.11–1.21)	0.097	0.65(0.29–1.47)	0.3
CHOL	1.48(0.50–4.41)	0.48	1.79(0.64–5.02)	0.27	1.69(0.56–5.06)	0.35	0.52(0.18–1.53)	0.24	0.60(0.21–1.71)	0.34
CESC	2.56(1.26–5.20)	**0.01**	–	–	3.29(1.83–5.92)	**<0.001**	0.38(0.21–0.69)	**<0.001**	0.57(0.31–1.03)	0.063
BLCA	1.52(0.98–2.34)	0.062	0.80(0.51–1.26)	0.34	2.87(1.56–5.28)	<0.001	0.46(0.26–0.83)	**0.01**	0.77(0.51–1.17)	0.22
**OVERALL**	1.60(1.34–1.92)	**<0.001**	1.12(0.90–1.40)	0.33	2.63(2.18–3.17)	**<0.001**	0.62(0.49–0.77)	**<0.001**	0.72(0.60–0.86)	**<0.001**

-: HRs can't be calculated, because there is only one gender group, or no one dies in one group.

UVM, Uveal Melanoma; UCEC, Uterine Corpus Endometrial Carcinoma; UCS, Uterine Carcinosarcoma; THYM, Thymoma; SKCM, Skin Cutaneous Melanoma; READ, Rectum adenocarcinoma; DLBC, Lymphoid Neoplasm Diffuse Large B-cell Lymphoma; LIHC, Liver hepatocellular carcinoma; KIRP, Kidney renal papillary cell carcinoma; ESCA, Esophageal carcinoma; COAD, Colon adenocarcinoma; CHOL, Cholangiocarcinoma; CESC, Cervical squamous cell carcinoma and endocervical adenocarcinoma; BLCA, Bladder Urothelial Carcinoma.

### Analyses of the cancer patients in each tumor type

Our above data showed that obesity might indicate a lower mortality in the whole population of cancer patients. Next we sought to find out the specific associations in different cancer types.

**Figure 2:**
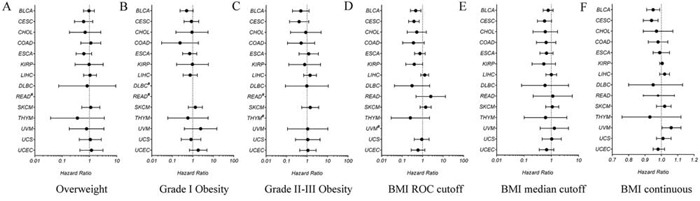
Hazard ratios for all-cause mortality relative to normal weight in different BMI groups in each cancer type **A.** overweight versus normal weight; **B.** Grade 1 obesity versus normal weight; **C.** Grade 2 and Grade 3 obesity versus normal weight; **D.** high BMI value versus low BMI value, with the BMI cutoff determined by ROC curve; **E.** high BMI value versus low BMI value, with the BMI cutoff determined by median value; **F.** high BMI value versus low BMI value, with BMI as a continuous variable. UVM, Uveal Melanoma; UCEC, Uterine Corpus Endometrial Carcinoma; UCS, Uterine Carcinosarcoma; THYM, Thymoma; SKCM, Skin Cutaneous Melanoma; READ, Rectum adenocarcinoma; DLBC, Lymphoid Neoplasm Diffuse Large B-cell Lymphoma; LIHC, Liver hepatocellular carcinoma; KIRP, Kidney renal papillary cell carcinoma; ESCA, Esophageal carcinoma; COAD, Colon adenocarcinoma; CHOL, Cholangiocarcinoma; CESC, Cervical squamous cell carcinoma and endocervical adenocarcinoma; BLCA, Bladder Urothelial Carcinoma.

Unexpectedly, no significant associations were found in any of the tumor type by the log-rank test (data not shown) or cox regression analysis (Figure 2A–2C). However, when we set the BMI value as a binary categorical variable, patients with higher BMI values (cutoff by the ROC curve, not by the median value) of BLCA and CESC showed significantly lower mortality (Figure 2D–2E). On the other hand, when we set the BMI as a continuous variable, patients with higher BMI values of BLCA and CESC also showed significantly lower mortality (Figure 2F).

This relationship was also verified by the cox regression analysis, which demonstrated that higher BMI value was correlated with lower mortality in CESC and BLCA (*P* < 0.05, Table 3 ). In addition, cox regression analysis also showed that some other variables, such as age and tumor stage, were also correlated with mortality in some cancer types (Table 3).

## DISCUSSION

In this study, we systemically report that postdiagnosis obesity might indicate a better prognosis in cancer patients. The association of obesity and cancer risk has been extensively explored. The mechanisms included insulin resistance and resultant chronic hyperinsulinaemia, increased bioavailability of steroid hormones, and localized chronic inflammation [8–12]. However, it is still controversial with respect to cancer motality.

Calle *et al*. reported that obesity was a negative prognosis factor for cancer by analyzing the largest cohort of patients in the US [13]. Reeves *et al*. provided similar results through the analysis in the UK [14]. These studies assessed the BMI before cancer development, leading to the popular perspective that prediangosis obesity indicated poor prognosis [15, 16]. However, discrepancies emerged when Schlesinger *et al*. indicated a decreased mortality risk among overweight colorectal cancer survivors (HR=0.79, 95%CI, 0.71-0.88, *P* < 0.05). We realized that they assessed the BMI after cancer development, which is consistent with our study (HR=0.64, 95%CI, 0.51-0.81, *P* < 0.001) [17].

Our results supported the survival advantage conferred by obesity where energy balance is likely to be negative[18, 19]. It's not hard to imagine that cachexia might indicate a poor prognosis[20]. However, when the patient suffers obesity before the development of cancer, side effects such as diabetes and cardiovascular diseases might have more impact on survial, which explains the results by Calle *et al.* and Reeves *et al.* In our study, besides the whole cancer population, only cervical cancer and bladder cancer showed lower mortality in patients with higher BMI values. We speculate that, the sample size is relatively large (>250) in cervical cancer and bladder cancer in our study, which might improve the statistical power in these patients. On the other hand, due to some hidden mechanisms, maybe the negative energy balance is more likely to affect the survival in these cancer types.

There are some limitations in our studies. Firstly, these findings should be interpreted cautiously because of small sample size. Secondly, we don't have the postdiagnosis weight loss data, which is a better evidence to demonstrate the impact of large energy store on cancer mortality. In conclusion, our data demonstrate that postdiagnosis obesity might indicate a lower cancer mortality.

## MATERIALS AND METHODS

### Patient samples

Clinical and survival data for 2670 cancer patients from 14 tumor types were obtained from the TCGA data portal (https://tcga-data.nci.nih.gov/tcga/) on July 24, 2015. The study was approved by the TCGA project manager and the Institutional Review Board of the Xi'an Jiaotong University.

### Clinical and follow-up data collection

Patient clinical data, including age, gender, height, weight and tumor stage were collected. Follow-up data such as vital status and survival time were also recoreded. We only included the patients with available BMI data.

### Statistical analysis

All data were analyzed by the R version 3.2.0 software and the SPSS version 19.0 software (SPSS, Chicago, IL, United States). Survival curves were constructed by the Kaplan-Meier method and compared by the log-rank test. The BMI cutoff points were determined by the receiver operating characteristic (ROC) curve analysis. Cox regression analysis was performed to evaluate the prognostic significance of the variables. Results are given as mean ± SD. *P* < 0.05 was considered statistically significant.
